# Impact of surface structuring on antibiotic elution from bone cement in two-stage revision for periprosthetic joint infection

**DOI:** 10.1186/s12891-026-10454-3

**Published:** 2026-09-12

**Authors:** Kevin Knappe, Mareike Schonhoff, Astrid Schenker, Raphael Trefzer, Jan Philippe Kretzer, Sebastian Jaeger

**Affiliations:** 1 Department of Orthopaedics, Heidelberg University Hospital, Heidelberg, 69118 Germany; 2 Research Center of Biomechanics and Implant Technology, Heidelberg University Hospital, Heidelberg, 69118 Germany

**Keywords:** Periprosthetic joint infections, PJI, Two-stage revision, Spacer, Antibiotic-loaded bone cement, Gentamicin, Vancomycin

## Abstract

**Purpose:**

Periprosthetic joint infection (PJI) is a serious complication after total joint arthroplasty and the leading cause of revision surgery. Two-stage revision with prosthesis removal, radical debridement, and interim antibiotic-loaded bone cement (ALBC) spacers is the current gold standard. ALBC spacers provide high local antibiotic concentrations and maintain joint space, with articulating designs improving interim mobility. Antibiotic release depends on cement composition, antibiotic content, and surface area, but the impact of surface structure remains unclear. This study investigated how surface morphology and area affect gentamicin and vancomycin release from ALBC in vitro.

**Methods:**

Copal G + V cement (2 g vancomycin, 0.5 g gentamicin per 43 g) was cast into standardized rectangular blocks according to DIN ISO 5833. A reference group without surface structuring and three groups with progressively increasing surface area and depth were analyzed. After polymerization, specimens were incubated in phosphate-buffered saline at 37 °C. Antibiotic release was measured at defined intervals over six weeks using chemiluminescence immunoassay (CLIA).

**Results:**

Surface structuring resulted in a clear increase in cumulative antibiotic release, particularly for vancomycin, with a consistent relationship between surface enlargement and total elution. The greatest surface structuring led to the highest release levels. Gentamicin showed a similar but less pronounced response, with a notable increase only at the highest degree of surface enlargement. The initial burst release contributed substantially to overall elution, while later-phase release differences between groups were less marked.

**Conclusion:**

Surface modification of PMMA cement influenced antibiotic elution in this in vitro experimental model, with increased surface depth resulting in enhanced vancomycin release. Gentamicin elution was less affected by surface geometry and showed significant improvement only with the greatest surface enlargement. These findings indicate that structured cement surfaces may influence local antibiotic release characteristics under controlled laboratory conditions. However, the clinical relevance of these surface modifications remains to be determined, as this study was limited to cement blocks incubated in phosphate-buffered saline and did not include a spacer, infection, or in vivo model. Further experimental and in vivo investigations are required to assess whether surface structuring can improve antibiotic delivery in clinically relevant settings.

**Supplementary Information:**

The online version contains supplementary material available at https://doi.org/10.1186/s12891-026-10454-3.

## Introduction

PJI is one of the most serious complications following total joint arthroplasty and represents the leading cause of revision surgery, as confirmed by the 2024 Annual Report of the American Joint Replacement Registry [1]. The current gold standard in the management of chronic PJI is a two-stage revision procedure [2–5]. During the first stage, the prosthesis and any residual bone cement are removed, followed by radical debridement of infected bone and soft tissue. This is typically supported by the use of antiseptic solutions and high-volume irrigation through a pulsatile lavage system.

For interim local antibiotic therapy, the use of ALBC spacers is well established (Supplementary Fig. 1). After an interval of six to twelve weeks, a second debridement is performed, and the definitive prosthesis is implanted [6, 7]. It has been shown that adding antibiotics intraoperatively to the bone cement can significantly increase local drug concentrations and improve infection control [8–11]. Furthermore, ALBC spacers help maintain joint space and soft tissue tension, and articulating designs can provide improved mobility and higher patient satisfaction during the interim period [12–14].

A sustained high local antibiotic concentration during the interim period is desirable to ensure effective eradication of pathogens while minimizing systemic toxicity. The release kinetics of antibiotics from bone cement depend on several factors, including the type and concentration of the incorporated antibiotics, the cement matrix, and critically, the surface area available for elution [9, 15–17]. An increased surface area can theoretically enhance the diffusion of antibiotics from the cement matrix by providing more interface for elution, especially during the initial burst release phase. However, the practical relevance of surface structuring on antibiotic release kinetics has not been thoroughly investigated.

The present study aimed to investigate, in an in vitro setting, how modifications of the surface morphology and surface area affect the release profiles of gentamicin and vancomycin from ALBC.

## Materials and methods

In this biomechanical in vitro study, Copal G + V (Heraeus Medical, Wehrheim, Germany) a commercially available antibiotic-loaded bone cement, containing 2 g of vancomycin and 0.5 g of gentamicin per 43 g of cement, was used. The composition for a 43 g batch includes: 43 g of powder consisting of 78% PMMA copolymer, 14% zirconium dioxide, 1% benzoyl peroxide, 2% gentamicin sulfate, and 5% vancomycin hydrochloride. The accompanying 20 mL of liquid consists of 98% methyl methacrylate and 2% N, N-dimethyl-p-toluidine. Other components include a chlorophyll–copper complex (colorant E141). The product was used as supplied by the manufacturer [18].

First, a reference group without surface structuring was prepared. The mold was designed to produce seven standardized rectangular blocks measuring 3.3 × 10 × 75 mm for each experimental group, in accordance with DIN ISO 5833. This well-established method has been published multiple times previously [19, 20].

**Fig. 1:**
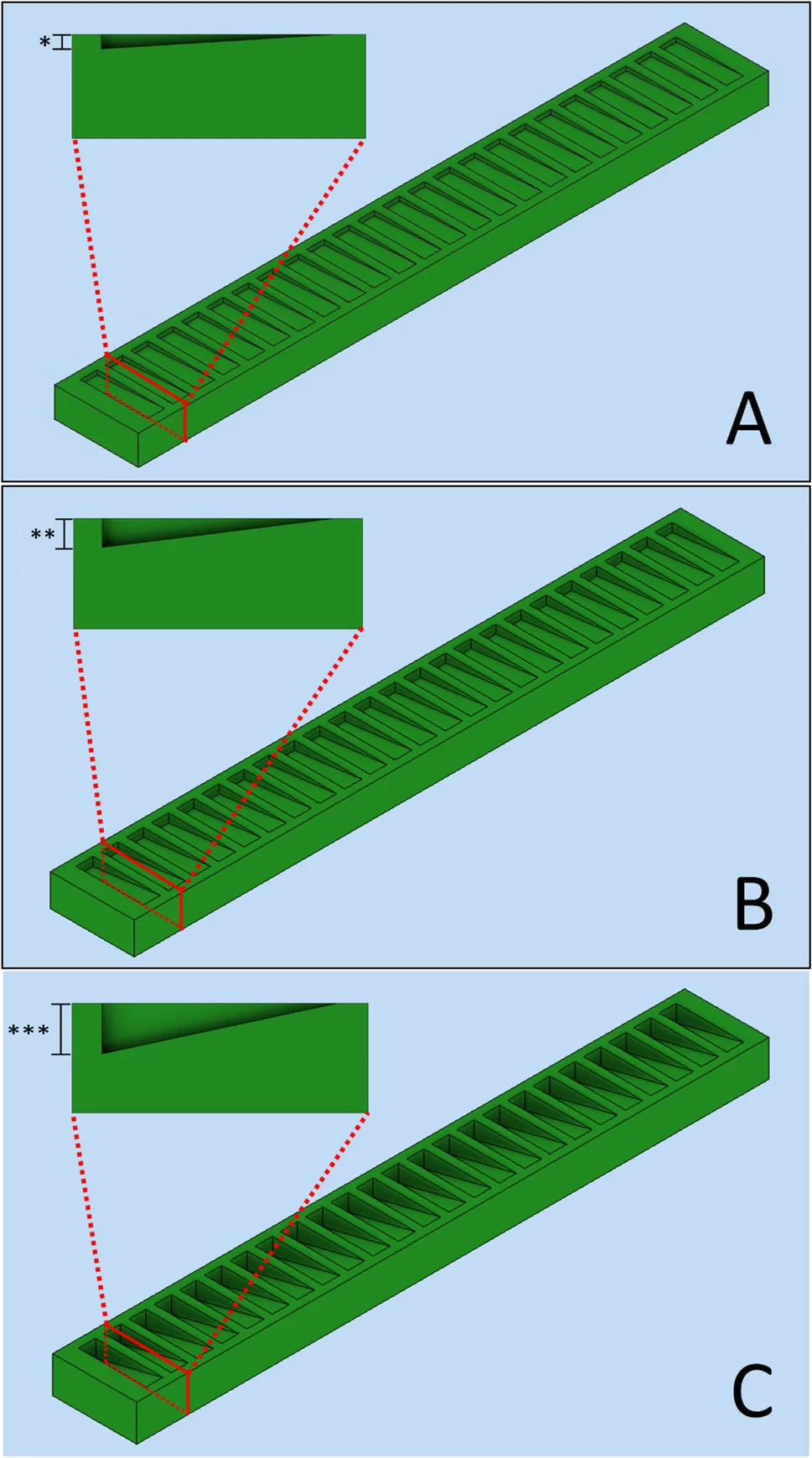
Cement blocks with three different surface structure depths. **A**: Surface group 1 with a structural depth of 0.5 mm (*). **B**: Surface group 2 with a structural depth of 1.0 mm (**). **C**: Surface group 3 with a structural depth of 2.0 mm (***)

**Fig. 2:**
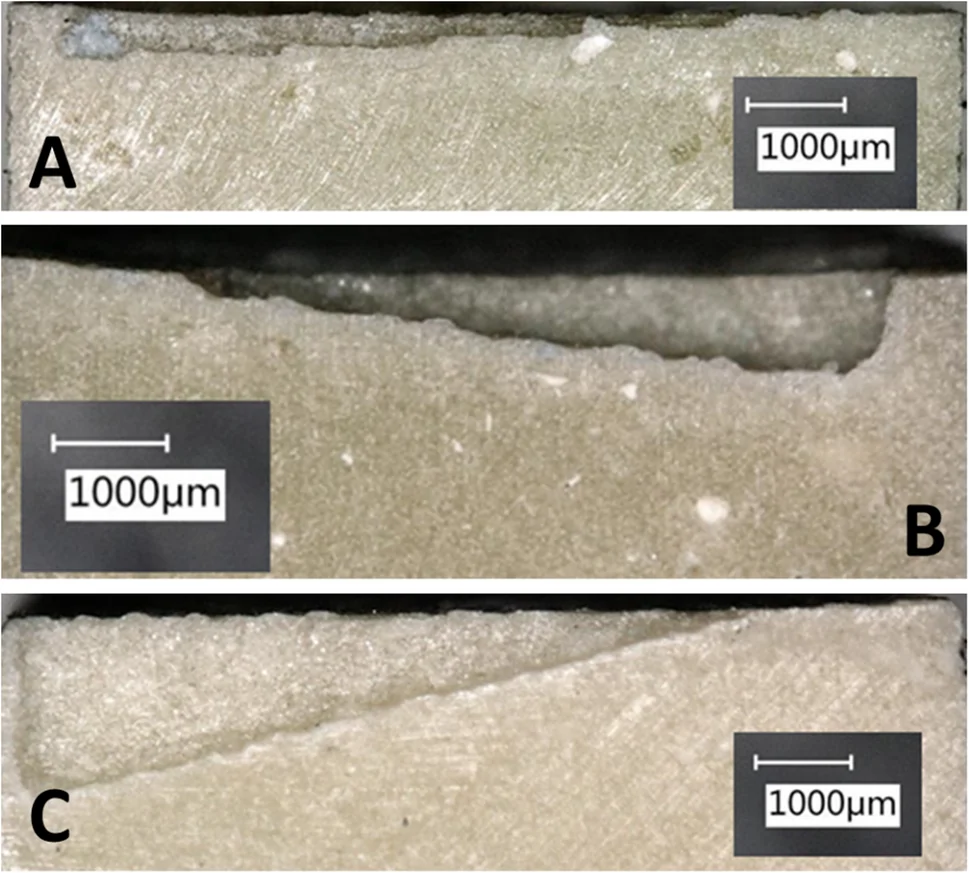
Cross-sectional images of the different surface configurations. **A**: Surface group 1 with a structural depth of 0.5 mm. **B**: Surface group 2 with a structural depth of 1.0 mm. **C**: Surface group 3 with a structural depth of 2.0 mm

Second, cement blocks with three different surface structure depths were fabricated (Figs. 1 and 2). The amount of bone cement used for each cement block was identical to that of the reference group. The structural depths were 0.5 mm in Surface group 1 (corresponding to a 14% increase in surface area compared with the reference group), 1.0 mm in Surface group 2 (28% increase), and 2.0 mm in Surface group 3 (56% increase). Accordingly, the percentage increase in surface area doubled from Surface group 1 to group 2, and doubled again from Surface group 2 to group 3. Surface area calculations were based on the CAD geometry of the molds designed in Autodesk Inventor Professional 2021 (Autodesk Inc., San Rafael, CA, USA). The total cement specimen surface area was derived directly from the predefined geometric parameters of the corresponding mold design, including groove depth, width, spacing, and number of surface structures (Supplementary Table 1).

The bone cement was processed according to the manufacturer´s instructions. As is common practice in revision surgeries, the cement was mixed at room temperature without vacuum. Sixty seconds after the start of mixing, the cement was injected into a mold using a cement gun.

A priori sample size calculation determined a required sample size of five specimens per group. To compensate for potential manufacturing defects, seven specimens per group were fabricated. The cement specimens of all four groups were left in the mold for one hour to allow complete polymerization of the bone cement. Prior to biomechanical testing, specimens were screened using predefined exclusion criteria, including air inclusions and deviations from ISO-specified dimensions. After verification, five of the seven blocks of each specimen were individually stored in 40 mL of phosphate-buffered saline (PBS) with a pH of 7.4 and incubated at 37 °C.

Antibiotic release from all groups was evaluated at 10 predefined time points: 5 h, 1 day, 2 days, 4 days, 1 week, 2 weeks, 3 weeks, 4 weeks, 5 weeks, and 6 weeks. At each time point, the incubation medium (40 mL PBS) was completely replaced, and the cement specimens were transferred to fresh containers containing 40 mL PBS. The removed medium was collected for analysis. Gentamicin and vancomycin concentrations were immediately quantified in a certified laboratory using CLIA.

### Statistical analysis

Statistical analyses were performed using IBM SPSS Statistics (IBM Corp., Armonk, NY, USA). Descriptive statistics were calculated for all outcome variables and are presented as mean ± standard deviation (SD). A two-factor mixed-design analysis of variance (mixed ANOVA) was conducted with time (T1–T10) as the within-subject factor and surface treatment (reference group, surface groups 1–3) as the between-subject factor. Prior to inferential testing, assumptions for mixed ANOVA were systematically evaluated. Normality of residual distributions was assessed using the Shapiro-Wilk test. Sphericity of repeated measures was examined using Mauchly’s test; whenever the assumption of sphericity was violated, Greenhouse-Geisser correction was applied to adjust the degrees of freedom. Homogeneity of variances between groups was verified before analysis. Interaction effects between time and treatment were analyzed first. In the presence of significant interactions, simple main effects analyses were subsequently performed for the between-subject factor (treatment) at individual time points, followed by Bonferroni-adjusted post hoc comparisons. Simple main effects of the within-subject factor (time) were additionally evaluated within each treatment group using pairwise comparisons with adjustment for multiple testing. Statistical significance was set at *p* < 0.05.

## Results

### Vancomycin release

**Fig. 3:**
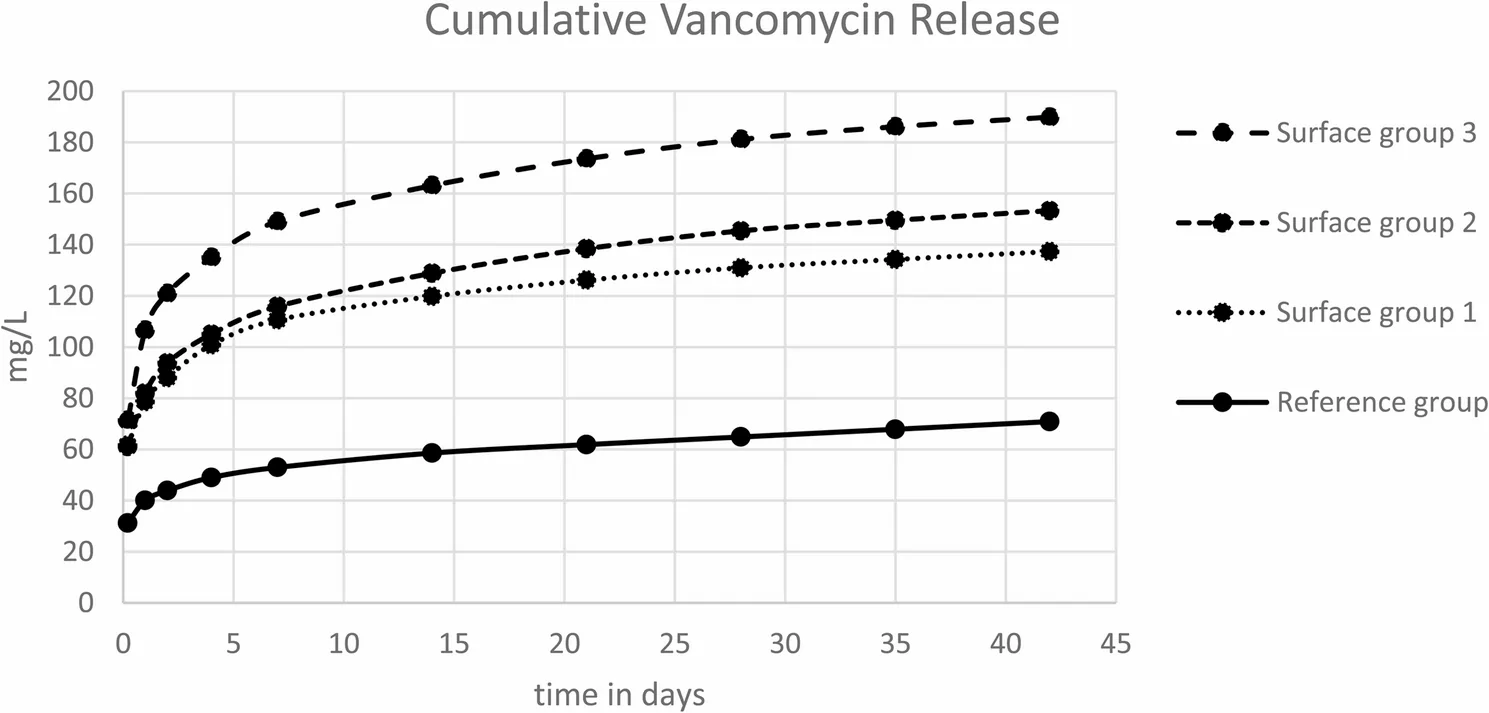
Cumulative Release of Vancomycin in mg/L at 10 predefined time points (5 h, 1 day, 2 days, 4 days, 1 week, 2 weeks, 3 weeks, 4 weeks, 5 weeks, and 6 weeks)

All structured surface groups (Surface groups 1–3) demonstrated increased cumulative vancomycin release over the 6-week observation period. Higher cumulative vancomycin release was observed in groups with predefined CAD-based surface modifications resulting in increased calculated surface area. The reference group consistently exhibited the lowest cumulative release across all time points. Repeated-measures analysis revealed a statistically significant interaction between time (T1–T10) and the investigated groups, demonstrating that the pattern of change over time differed significantly between groups (Greenhouse–Geisser correction: F(3.50, 18.680) = 70.23, *p* < 0.001). At each time point, the cumulative vancomycin release in all surface groups (1–3) was significantly higher than that of the reference group (Fig. 3, Tables 1 and 2).

**Table 1:** Mean Cumulative Release of Vancomycin in mg/L at 10 predefined time points: 0.208 days correspond to 5 h. Reported *p*-values are Bonferroni-adjusted to account for multiple comparisons

Days	0.208	1	2	4	7	14	21	28	35	42
Surface group 1 (mg/L) Mean ± SD	61.6 ± 3.6	78.7 ± 5.6	87.9 ± 6.2	100.8 ± 8.4	110.6 ± 9.5	119.8 ± 14.0	126.1 ± 14.1	130.9 ± 14.9	134.2 ± 15.2	137.3 ± 15.3
Reference group (mg/L) Mean ± SD	31.2 ± 0.7	40.0 ± 1.1	43.9 ± 0.9	49.0 ± 0.9	52.9 ± 0.8	58.5 ± 0.5	61.8 ± 0.4	64.8 ± 0.4	67.8 ± 0.4	70.8 ± 0.4
*p*-value	< 0.001	< 0.001	< 0.001	< 0.001	< 0.001	< 0.001	< 0.001	< 0.001	< 0.001	< 0.001
Surface group 2 (mg/L) Mean ± SD	61.1 ± 3.2	82.0 ± 2.6	93.8 ± 2.9	105.0 ± 3.9	115.9 ± 4.9	128.8 ± 5.8	138.4 ± 6.6	145.4 ± 7.3	149.5 ± 7.6	153.3 ± 8.5
Reference group (mg/L) Mean ± SD	31.2 ± 0.7	40.0 ± 1.1	43.9 ± 0.9	49.0 ± 0.9	52.9 ± 0.8	58.5 ± 0.5	61.8 ± 0.4	64.8 ± 0.4	67.8 ± 0.4	70.8 ± 0.4
*p*-value	< 0.001	< 0.001	< 0.001	< 0.001	< 0.001	< 0.001	< 0.001	< 0.001	< 0.001	< 0.001
Surface group 3 (mg/L) Mean ± SD	71.3 ± 4.8	106.6 ± 6.3	120.9 ± 6.9	135.0 ± 7.1	149.0 ± 7.6	163.1 ± 7.7	173.5 ± 7.8	181.1 ± 8.2	186.1 ± 8.3	189.8 ± 9.4
Reference group (mg/L) Mean ± SD	31.2 ± 0.7	40.0 ± 1.1	43.9 ± 0.9	49.0 ± 0.9	52.9 ± 0.8	58.5 ± 0.5	61.8 ± 0.4	64.8 ± 0.4	67.8 ± 0.4	70.8 ± 0.4
*p*-value	< 0.001	< 0.001	< 0.001	< 0.001	< 0.001	< 0.001	< 0.001	< 0.001	< 0.001	< 0.001

**Table 2:** Eluate concentrations of Vancomycin in mg/L at 10 predefined time points (5 h, 1 day, 2 days, 4 days, 1 week, 2 weeks, 3 weeks, 4 weeks, 5 weeks, and 6 weeks)

Days	0.208	1	2	4	7	14	21	28	35	42
Reference group (mg/L) Mean ± SD	31.2 ± 0.7	8.8 ± 0.6	3.9 ± 0.3	5.1 ± 0.2	4.0 ± 0.3	5.6 ± 0.4	3.3 ± 0.2	3.0 ± 0.0	3.0 ± 0.0	3.0 ± 0.0
Surface group 1 (mg/L) Mean ± SD	61.6 ± 3.6	17.1 ± 2.2	9.3 ± 1.0	12.9 ± 2.3	9.8 ± 1.1	11.5 ± 2.1	6.3 ± 0.4	4.8 ± 0.8	3.3 ± 0.3	3.1 ± 0.1
Surface group 2 (mg/L) Mean ± SD	61.1 ± 3.2	20.9 ± 1.0	11.8 ± 1.8	11.3 ± 1.2	10.8 ± 1.5	12.9 ± 1.0	9.6 ± 1.0	7.0 ± 1.1	4.1 ± 0.5	3.8 ± 1.3
Surface group 3 (mg/L) Mean ± SD	71.3 ± 4.8	35.2 ± 3.4	14.3 ± 1.2	14.0 ± 1.2	14.1 ± 0.8	14.0 ± 1.3	10.5 ± 0.3	7.6 ± 0.4	5.0 ± 0.2	3.7 ± 1.3

Following the identification of a significant interaction effect across the measurement time points, simple main effects of the between-subject factor were examined. Pairwise comparisons between groups were performed using Bonferroni-adjusted post hoc tests. The results are presented in Table 1. The analysis revealed a statistically significant effect of time (T1–T10) on antibiotic release, indicating significant changes in release over the measurement period (Reference Group (F(9,36) = 7401.10, *p* < 0.01); Surface group 1 (F(9,36) = 142.55, *p* < 0.01); Surface group 2 (F(9,36) = 674.51, *p* < 0.01); Surface group 3 (F(9,36) = 1912.07, *p* < 0.01). After 6 weeks, cumulative vancomycin release was elevated by 94% in Surface group 1 (*p* < 0.001), by 117% in Surface group 2 (*p* < 0.001), and by 168% in Surface group 3 (*p* < 0.001) compared with the reference group.

The individual mass release values per specimen (mg/specimen) for each measurement time point are additionally provided in tabulated form in the Supplementary Material (Supplementary Table 2).

### Gentamicin release

**Fig. 4:**
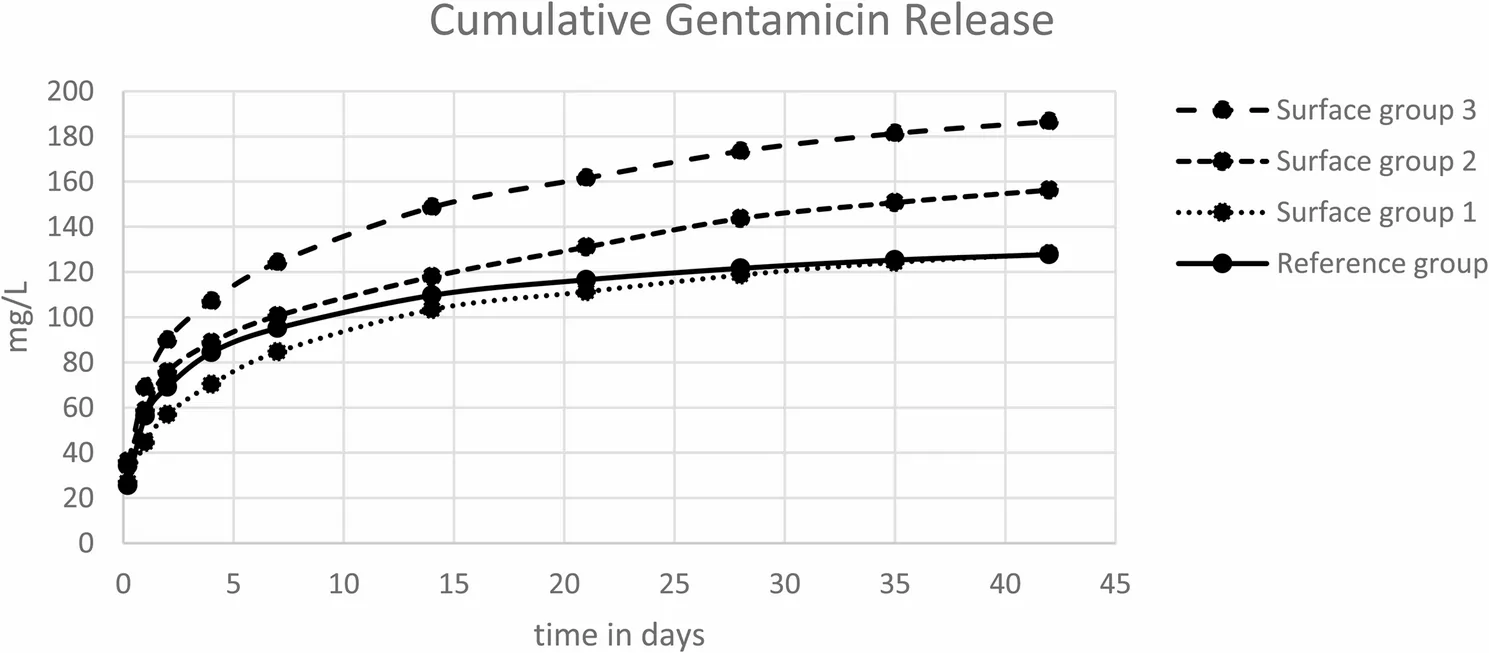
Cumulative Release of Gentamicin in mg/L at 10 predefined time points (5 h, 1 day, 2 days, 4 days, 1 week, 2 weeks, 3 weeks, 4 weeks, 5 weeks, and 6 weeks)

A similar pattern was observed for gentamicin release. Surface groups 2 and 3 showed progressively higher cumulative release with increasing predefined CAD-based surface modifications and calculated surface area, whereas Surface group 1 did not differ significantly from the reference group. Repeated-measures analysis revealed a statistically significant interaction between time (T1–T10) and the investigated groups, demonstrating that the pattern of change over time differed significantly between groups (Greenhouse–Geisser correction: F(3.51, 18.74) = 8.35, *p* = 0.001) (Fig. 4, Tables 3 and 4).

**Table 3:** Mean Cumulative Release of Gentamicin in mg/L at 10 predefined time points: 0.208 days corresponds to 5 h. Reported *p*-values are Bonferroni-adjusted to account for multiple comparisons

Days	0.208	1	2	4	7	14	21	28	35	42
Surface group 1 (mg/L) Mean ± SD	27.1 ± 2.7	44.5 ± 4.4	57.0 ± 6.8	70.3 ± 9.5	84.6 ± 12.4	103.4 ± 17.2	111.3 ± 19.3	118.7 ± 20.3	124.2 ± 21.7	128.0 ± 22.5
Reference group (mg/L) Mean ± SD	25.6 ± 6.9	56.3 ± 3.4	69.1 ± 4.6	84.3 ± 5.4	95.1 ± 6.0	109.7 ± 9.2	116.6 ± 10.6	121.6 ± 11.6	125.3 ± 12.5	127.7 ± 12.7
*p*-value	1.0	0.032	0.112	0.164	0.810	1.0	1.0	1.0	1.0	1.0
Surface group 2 (mg/L) Mean ± SD	36.2 ± 4.7	58.7 ± 4.3	75.8 ± 6.4	88.8 ± 6.7	100.6 ± 6.8	117.9 ± 9.3	131.0 ± 12.2	143.7 ± 14.7	150.7 ± 16.0	156.3 ± 16.9
Reference group (mg/L) Mean ± SD	25.6 ± 6.9	56.3 ± 3.4	69.1 ± 4.6	84.3 ± 5.4	95.1 ± 6.0	109.7 ± 9.2	116.6 ± 10.6	121.6 ± 11.6	125.3 ± 12.5	127.7 ± 12.7
*p*-value	0.068	1.0	1.0	1.0	1.0	1.0	0.867	0.250	0.168	0.107
Surface group 3 (mg/L) Mean ± SD	34.2 ± 7.7	68.9 ± 9.1	89.8 ± 10.3	107.1 ± 13.0	124.2 ± 14.4	148.7 ± 15.4	161.6 ± 16.1	173.4 ± 15.2	181.3 ± 14.8	186.6 ± 14.5
Reference group (mg/L) Mean ± SD	25.6 ± 6.9	56.3 ± 3.4	69.1 ± 4.6	84.3 ± 5.4	95.1 ± 6.0	109.7 ± 9.2	116.6 ± 10.6	121.6 ± 11.6	125.3 ± 12.5	127.7 ± 12.7
*p*-value	0.198	0.018	0.002	0.007	0.003	0.002	0.001	0.001	< 0.001	< 0.001

**Table 4:** Eluate concentrations of Gentamicin in mg/L at 10 predefined time points (5 h, 1 day, 2 days, 4 days, 1 week, 2 weeks, 3 weeks, 4 weeks, 5 weeks, and 6 weeks)

Days	0.208	1	2	4	7	14	21	28	35	42
Reference group (mg/L) Mean ± SD	25.6 ± 6.9	30.7 ± 4.7	12.8 ± 2.1	15.3 ± 2.0	10.8 ± 1.2	14.5 ± 3.4	6.9 ± 1.5	5.1 ± 1.1	3.7 ± 1.0	2.4 ± 0.3
Surface group 1 (mg/L) Mean ± SD	27.1 ± 2.7	17.5 ± 2.1	12.4 ± 3.1	13.3 ± 2.8	14.3 ± 3.0	18.8 ± 6.6	7.9 ± 2.1	7.4 ± 1.1	5.6 ± 1.9	3.8 ± 0.9
Surface group 2 (mg/L) Mean ± SD	36.2 ± 4.7	22.6 ± 1.4	17.0 ± 3.3	13.0 ± 1.9	11.8 ± 1.9	17.3 ± 4.1	13.1 ± 4.5	12.7 ± 3.8	7.0 ± 1.7	5.5 ± 1.2
Surface group 3 (mg/L) Mean ± SD	34.2 ± 7.7	34.7 ± 4.1	20.9 ± 1.7	17.3 ± 3.1	17.1 ± 2.1	24.4 ± 3.2	12.9 ± 1.2	11.9 ± 2.1	7.9 ± 1.3	5.3 ± 0.5

Following the identification of a significant interaction effect across the measurement time points, simple main effects of the between-subject factor were examined. Pairwise comparisons between groups were performed using Bonferroni-adjusted post hoc tests. The results are presented in Table 3. The analysis revealed a statistically significant effect of time (T1–T10) on antibiotic release, indicating significant changes in release over the measurement period (Reference Group (F(9,36) = 199.93, *p* < 0.01); Surface group 1 (F(9,36) = 102.30, *p* < 0.01); Surface group 2 (F(9,36) = 136.47, *p* < 0.01); Surface group 3 (F(9,36) = 596.45, *p* < 0.01). After 6 weeks, cumulative gentamicin release was higher by 22% in Surface group 2 (*p* = 0.107) and by 46% in Surface group 3 (*p* < 0.001) compared to the reference group, while no difference was observed for Surface group 1 (*p* = 1.00).

The individual mass release values per specimen (mg/specimen) for each measurement time point are additionally provided in tabulated form in the Supplementary Material (Supplementary Table 3).

## Discussion

In this study, structured PMMA cement surfaces increased cumulative antibiotic release compared with unstructured reference specimens. Vancomycin release was significantly enhanced in all surface-modified groups and showed progressively higher release with predefined CAD-based surface modifications resulting in increased calculated surface area, resulting in up to 168% higher cumulative release after 6 weeks. Gentamicin demonstrated a similar trend, although the effect was less pronounced, with significant improvement only in the group with the greatest surface enlargement.

Although it is clear that bone cement can be loaded with antibiotics, which are then effectively released from the cement [21–23], varying data on the actual efficacy of antibiotic-loaded bone cements with regard to the prevention of postoperative infections in primary arthroplasty exist [24–27]. Aminoglycosides such as gentamicin exhibit their primary antimicrobial activity against Gram-negative organisms. In clinical practice, they are frequently combined with vancomycin, whose spectrum is largely restricted to Gram-positive pathogens, resulting in pronounced synergistic effects. For gentamicin, achieving a high peak concentration relative to the minimal inhibitory concentration is considered essential for optimal bactericidal efficacy [28]. Systemically, however, serum levels exceeding approximately 1 mg/L have been associated with an increased risk of nephrotoxicity and even higher mortality rates [29–31]. Although local antibiotic application is often viewed as safer, isolated cases of renal impairment following implantation of antibiotic-loaded bone cement have also been reported [32]. Current recommendations therefore advise maintaining systemic gentamicin concentrations below 0.5-1.0 mg/L [33]. Similar concerns have been raised regarding vancomycin-loaded cement, with reports of renal dysfunction in this context as well [34, 35].

These observations contrast with data from intraoperative intra-articular vancomycin administration, in which extremely high local concentrations were measured while systemic exposure remained negligible. In one study, vancomycin levels in wound drainage fluid reached mean values of approximately 550 mg/L on the first postoperative day and remained around 80 mg/L on day five, whereas serum concentrations consistently stayed below the assay’s detection threshold of 2.0 mg/L [36]. Comparable findings have been described in additional investigations demonstrating high local but minimal systemic vancomycin levels after local application [37, 38]. The same pharmacokinetic pattern has long been documented for gentamicin: intra-articular administration results in high joint concentrations with only low systemic and urinary levels [39], and similar behaviour is observed following local application into bone tissue [40]. Taken together, these and other reports suggest that local delivery of gentamicin or vancomycin is generally not associated with clinically relevant systemic toxicity [41].

Defining universally applicable intra-articular target concentrations remains challenging, as effective antibiotic levels depend strongly on the causative pathogen and its specific MIC. In periprosthetic joint infections of the hip and knee, coagulase-negative staphylococci - particularly Staphylococcus epidermidis and Staphylococcus aureus account for the majority of cases. Coagulase-negative staphylococci are implicated in approximately 30–43% of infections, whereas S. aureus is responsible for roughly 12–24% [42–45]. For vancomycin, reported MIC values for S. aureus typically approximate 1 mg/L [46, 47]. Gentamicin demonstrates bactericidal activity against S. aureus at MIC values of around 0.125–0.25 mg/L [48]. To minimize the emergence of resistant bacterial subpopulations, aminoglycosides usually need to reach peak concentrations roughly an order of magnitude above the MIC [49]. Accordingly, gentamicin concentrations of approximately 2 mg/L are considered therapeutically effective [28].

Several systematic reviews have addressed the clinical use of intravenous vancomycin, including dosing principles, monitoring strategies, and pharmacokinetic considerations [46, 50–52]. If one assumes a minimal inhibitory concentration of approximately 3.0 mg/L as the lower threshold for effective vancomycin activity- reflecting the lower limit of detection in the respective analyses- this concentration is not achieved with Reference group at the time points 8–10. Under these conditions, the additional clinical benefit of vancomycin supplementation in this specific cement formulation appears questionable. However, the temporal dynamics of antibiotic concentrations in vivo remain insufficiently understood, and it is uncertain how these levels evolve after implantation.

Moreover, it cannot be conclusively determined whether the comparatively low antibiotic concentrations released from certain cement formulations are adequate for the effective eradication of bacterial pathogens. Although polymethylmethacrylate (PMMA) can absorb approximately 2% of its weight in fluid [34], the material’s intrinsically dense and hydrophobic structure limits extensive fluid penetration. Recent evidence has demonstrated a strong correlation between cement porosity- enhanced by higher antibiotic loading and increased radiopacifier content- and the elution kinetics of gentamicin [53]. According to these findings, greater porosity may facilitate higher fluid absorption and thus promote enhanced antibiotic washout.

The predefined CAD-based surface modifications and corresponding depth variations across the structured groups were associated with a progressive increase in vancomycin release. These differences were significant for all vancomycin groups and across all time points. Assuming that antibiotic elution predominantly occurs from the outermost cement layer [54], the observed association in our experiments appears consistent with this principle.

In contrast, evaluation of cumulative gentamicin release revealed nearly identical elution profiles for the reference group and Surface group 1. Likewise, statistical analysis showed a significantly increased gentamicin release only for Surface group 3 across 9 of the 10 measurement intervals. A potential explanation may lie in the molecular characteristics of the antibiotics: vancomycin exhibits approximately threefold greater molecular size and substantially higher molecular weight than gentamicin. Gentamicin, as the smaller molecule, exhibits a faster and higher release from bone cement compared with vancomycin [55]. Moreover, several experimental studies have demonstrated that the cumulative release of gentamicin from PMMA cement is markedly higher than that of vancomycin during the initial days and weeks [56]. This is already reflected in the fact that the commercially available revision cement contains four times more vancomycin than gentamicin (2 g versus 0.5 g). Our observations suggest that the deepened surface structures with increased calculated surface area were associated with significant changes only once a certain threshold was exceeded (Surface group 3 with a depth of 2 mm and a 56% increase in surface area).

Although antibiotic release is known to peak within the first days as a so-called initial burst release, the isolated analysis of the extended period from day 7 to day 42 also demonstrated markedly higher release values for vancomycin. As previously noted, this effect was considerably less pronounced for gentamicin. It must also be considered that this later phase was assessed at wider intervals (weekly), reflecting the longer observation period. A saturation effect of the buffer solution appears unlikely, given that substantially higher antibiotic concentrations were already detected at the initial 5-hour time point.

Even though this work does not explain why antibiotic release from ALBC is not proportional for both antibiotics at all time points, this analysis provides experimental evidence that predefined increases in cement surface structuring and calculated surface area are associated with higher antibiotic concentrations in the eluate.

Assuming that specific modifications of the surface structure of antibiotic-loaded bone cement can lead to increased local antibiotic release, a potential direct clinical benefit may be derived. By mechanically modifying the cement during its still malleable, polymerising phase, the surface could be structured using simple tools such as patterned plates or a roller-type device with a defined surface texture. This approach may enable controlled modification of cement surface geometry and potentially influence local antibiotic release characteristics.

### Limitation

This investigation was conducted entirely in vitro, meaning that important physiological processes such as metabolism, fluid circulation, and - particularly relevant in the context of articulating spacers - dynamic motion of tissues and implant components were absent. Consequently, the experimental conditions only approximate, but do not replicate, the in vivo environment.

Another limitation arises from the in vitro study design using a static PBS model with a fixed elution volume (40 mL). This setup does not replicate physiological in vivo conditions, which are characterized by continuous fluid turnover, protein binding, shear stress, and mechanical loading. It therefore remains uncertain to what extent the observed elution kinetics accurately reflect the clinical situation in vivo. In addition, the ratio between specimen geometry and elution medium may differ substantially from physiological conditions, potentially influencing antibiotic release behavior. A further limitation relates to the analytical method used for antibiotic quantification. The CLIA assays for vancomycin and gentamicin are primarily validated for clinical samples, particularly serum matrices. In the present study, these assays were applied to PBS samples using the same analytical procedures and measurement conditions as established for serum analysis. Although this approach allows comparative assessment between experimental groups under identical conditions, a separate PBS-specific validation including matrix effect evaluation, spike-recovery testing, and dilution verification was not performed. Therefore, the absolute measured concentrations should be interpreted with caution, and the results should primarily be considered in terms of relative differences in antibiotic elution between the tested cement configurations. Future studies should include matrix-specific assay validation to confirm the accuracy of antibiotic quantification in elution models.

Additionally, the study focused exclusively on quantifying antibiotic concentrations without assessing the corresponding antibacterial activity, which limits direct conclusions regarding biological efficacy. No microbiological assays were performed to evaluate the actual bioactivity of the eluted agents. Therefore, it remains unclear to what extent the released antibiotics retain their full antimicrobial efficacy after incorporation into and elution from bone cement. Accordingly, any interpretation of the MIC values and their clinical implications should be made with caution. Future investigations, including agar diffusion assays or other microbiological inhibition tests, should be considered to address this aspect more comprehensively. The interpretation of clinical relevance is further complicated by the absence of robust data on therapeutic intra-articular antibiotic levels in periprosthetic joint infection, as standardized large-scale clinical trials are still lacking. Accordingly, the discussion of MIC values and proposed target concentrations for vancomycin and gentamicin remains necessarily speculative. Any extrapolation from systemic pharmacokinetic data or MIC-derived thresholds to local antibiotic concentrations released from spacers must therefore be regarded as hypothetical and interpreted with caution.

Moreover, the present experimental design does not include mechanical testing and therefore does not allow any conclusions regarding the mechanical stability, fatigue resistance, wear behavior, debris generation, or fracture risk of surface-structured PMMA cement spacers. The assumption that superficial surface modifications of up to 2 mm may have limited mechanical relevance is based solely on a theoretical consideration related to the use of temporary, non-articulating spacers (e.g., arthrodesis spacers), which are typically exposed to limited mechanical demands. However, no experimental data were obtained to verify the mechanical integrity of modified spacers, and these considerations cannot be transferred to articulating or load-bearing spacer designs. Furthermore, it must be acknowledged that increasing the depth of the surface structures also resulted in increased calculated surface area. Therefore, within the present experimental setup, it was not possible to evaluate these two parameters independently.

Despite these limitations, the chosen setup - designed specifically to compare how alterations in cement morphology, surface area, and surface depth affect antibiotic elution - offers a high degree of reproducibility and methodological standardization, allowing meaningful comparisons between the tested configurations.

## Conclusion

Surface modification of PMMA cement influenced antibiotic elution in this in vitro experimental model, with increased surface depth resulting in enhanced vancomycin release. Gentamicin elution was less affected by surface geometry and showed significant improvement only with the greatest surface enlargement. These findings indicate that structured cement surfaces may influence local antibiotic release characteristics under controlled laboratory conditions. However, the clinical relevance of these surface modifications remains to be determined, as this study was limited to cement blocks incubated in phosphate-buffered saline and did not include a spacer, infection, or in vivo model. Further experimental and in vivo investigations are required to assess whether surface structuring can improve antibiotic delivery in clinically relevant settings.

## Supplementary Information

Supplementary Material 1. Supplementary Figure 1: Preoperative radiographs demonstrating septic loosening of a total knee arthroplasty in a 59-year-old male patient (left). Postoperative radiograph after prosthesis removal and implantation of an arthrodesis bone cement spacer, additionally using two carbon fiber rods and a connection clamp (right). Radiographic images were retrieved from the institutional picture archiving and communication system (PACS). The images were anonymized and used solely for illustrative purposes. Supplementary Table 1: CAD-derived surface characteristics of the cement specimens. Supplementary Table 2: Vancomycin mass released per specimen in mg/specimen, for each interval. Supplementary Table 3: Gentamicin mass released per specimen in mg/specimen, for each interval.

## Data Availability

The dataset used and/or analyzed during the current study is available from the corresponding author upon reasonable request.

## Abbreviations

ALBC: Antibiotic-loaded bone cement
CLIA: Chemiluminescence immunoassay
PACS: Picture archiving and communication system
PBS: Phosphate-buffered saline
PJI: Periprosthetic joint infection
PMMA: Polymethylmethacrylate
