# Supplementary material for: Impact of surface structuring on antibiotic elution from bone cement in two-stage revision for periprosthetic joint infection
Source: BMC Musculoskelet Disord. 2026 Sep 12;27:819. doi: 10.1186/s12891-026-10454-3 (PMC13637365; doi:10.1186/s12891-026-10454-3)
Supplement: Supplementary file 1 [file 12891_2026_10454_MOESM1_ESM.docx]

**Supplementary Material:**

Supplementary Figure 1: Preoperative radiographs demonstrating septic loosening of a total knee arthroplasty in a 59-year-old male patient (left). Postoperative radiograph after prosthesis removal and implantation of an arthrodesis bone cement spacer, additionally using two carbon fiber rods and a connection clamp (right). Radiographic images were retrieved from the institutional picture archiving and communication system (PACS). The images were anonymized and used solely for illustrative purposes.

*Supplementary Table 1: CAD-derived surface characteristics of the cement specimens.*

| Cement-structure | Surface area [mm²] | Volume [mm³] | Surface area-to-volume ratio | Surface area increase [%] |
| --- | --- | --- | --- | --- |
| Reference group | 2063,7 | 2478,3 | 0,83 | 100% |
| Surface group 1 | 2348,7 | 2478,3 | 0,95 | 114% |
| Surface group 2 | 2636,7 | 2478,3 | 1,06 | 128% |
| Surface group 3 | 3221,3 | 2478,3 | 1,30 | 156% |

*Supplementary Table 2:* *Vancomycin mass released per specimen in mg/specimen, for each interval.*

| **Days** | **0.208** | **1** | **2** | **4** | **7** | **14** | **21** | **28** | **35** | **42** |
| --- | --- | --- | --- | --- | --- | --- | --- | --- | --- | --- |
| **Reference group (mg)** | 1,25 | 0,35 | 0,15 | 0,20 | 0,16 | 0,22 | 0,13 | 0,12 | 0,12 | 0,12 |
| **Mean ± SD** | 0,03 | 0,02 | 0,01 | 0,01 | 0,01 | 0,02 | 0,01 | 0,00 | 0,00 | 0,00 |
|  | | | | | | | | | | |
| **Surface group 1 (mg)** | 2,46 | 0,68 | 0,37 | 0,52 | 0,39 | 0,46 | 0,25 | 0,19 | 0,13 | 0,12 |
| **Mean ± SD** | 0,14 | 0,09 | 0,04 | 0,09 | 0,04 | 0,22 | 0,01 | 0,03 | 0,01 | 0,01 |
|  | | | | | | | | | | |
| **Surface group 2 (mg)** | 2,44 | 0,84 | 0,47 | 0,45 | 0,43 | 0,52 | 0,38 | 0,28 | 0,17 | 0,15 |
| **Mean ± SD** | 0,13 | 0,04 | 0,07 | 0,05 | 0,06 | 0,04 | 0,04 | 0,04 | 0,02 | 0,05 |
|  | | | | | | | | | | |
| **Surface group 3 (mg)** | 2,85 | 1,41 | 0,57 | 0,56 | 0,56 | 0,56 | 0,42 | 0,30 | 0,20 | 0,15 |
| **Mean ± SD** | 0,19 | 0,13 | 0,05 | 0,05 | 0,03 | 0,05 | 0,01 | 0,02 | 0,01 | 0,05 |

*Supplementary Table 3: Gentamicin mass released per specimen in mg/specimen, for each interval.*

| **Days** | **0.208** | **1** | **2** | **4** | **7** | **14** | **21** | **28** | **35** | **42** |
| --- | --- | --- | --- | --- | --- | --- | --- | --- | --- | --- |
| **Reference group (mg)** | 1,02 | 1,23 | 0,51 | 0,61 | 0,43 | 0,58 | 0,28 | 0,20 | 0,15 | 0,10 |
| **Mean ± SD** | 0,28 | 0,19 | 0,09 | 0,08 | 0,05 | 0,13 | 0,06 | 0,05 | 0,04 | 0,01 |
|  | | | | | | | | | | |
| **Surface group 1 (mg)** | 1,08 | 0,70 | 0,50 | 0,53 | 0,57 | 0,75 | 0,31 | 0,30 | 0,22 | 0,15 |
| **Mean ± SD** | 0,11 | 0,08 | 0,12 | 0,11 | 0,12 | 0,26 | 0,08 | 0,04 | 0,08 | 0,04 |
|  | | | | | | | | | | |
| **Surface group 2 (mg)** | 1,45 | 0,90 | 0,68 | 0,52 | 0,47 | 0,69 | 0,53 | 0,51 | 0,28 | 0,22 |
| **Mean ± SD** | 0,19 | 0,06 | 0,13 | 0,08 | 0,08 | 0,17 | 0,18 | 0,15 | 0,07 | 0,05 |
|  | | | | | | | | | | |
| **Surface group 3 (mg)** | 1,37 | 1,39 | 0,83 | 0,69 | 0,68 | 0,98 | 0,52 | 0,48 | 0,31 | 0,21 |
| **Mean ± SD** | 0,31 | 0,16 | 0,07 | 0,12 | 0,09 | 0,13 | 0,05 | 0,09 | 0,05 | 0,02 |
